# Supplementary material for: Enzymatic Characterization of Wild-Type and Mutant Janus Kinase 1
Source: Cancers (Basel). 2019 Nov 1;11(11):1701. doi: 10.3390/cancers11111701 (PMC6896158; doi:10.3390/cancers11111701)
Supplement: Supplementary file 1 [file cancers-11-01701-s001.pdf]

Supplementary Figure S1

A

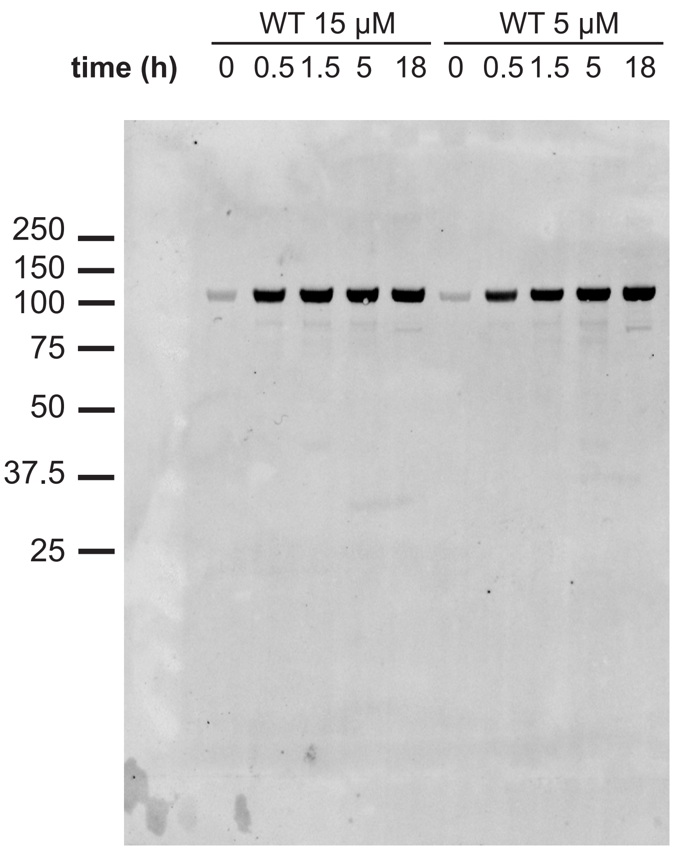

WB: pJAK1

From Figure 4A

Densitometry: 5.9; 11.6; 15.1; 15.1; 15.9; 4.3; 8.9; 12.8; 17.4; 19.3

B

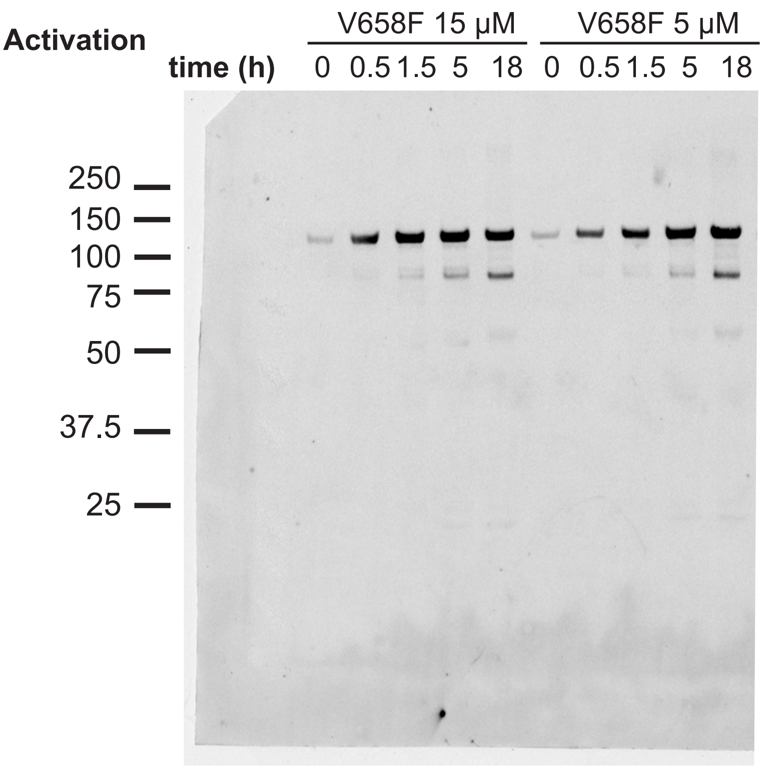

WB: pJAK1

From Figure 4A

Densitometry: 3.8; 7.0; 11.6; 13.0; 10.7; 3.2; 5.7; 8.1; 12.3; 13.5

C

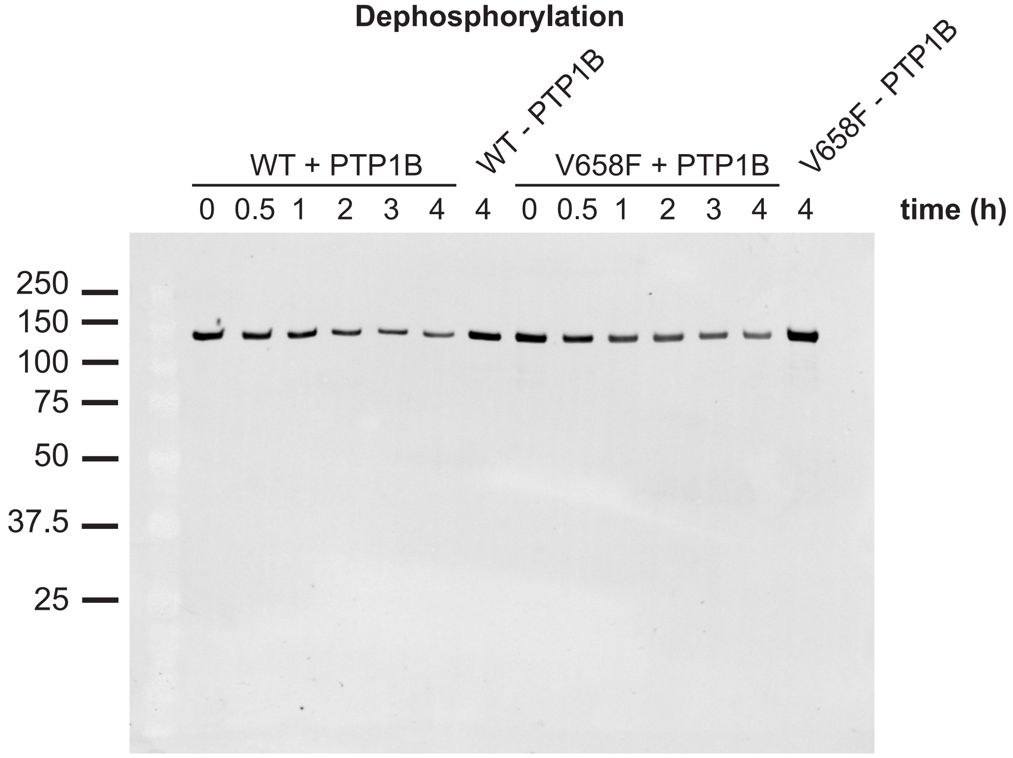

WB: pJAK1

From Figure 4B

Densitometry data: 19.6; 15.0; 14.4; 11.4; 10.1; 9.3; 21.1; 21.8; 15.6; 13.0; 13.1; 10.7; 9.7; 25.7
